# A Brief Overview of the Cerebrospinal Fluid System and Its Implications for Brain and Spinal Cord Diseases

**DOI:** 10.3389/fnhum.2021.737217

**Published:** 2022-01-21

**Authors:** Thea Overgaard Wichmann, Helle Hasager Damkier, Michael Pedersen

**Affiliations:** ^1^Department of Neurosurgery, Aarhus University Hospital, Aarhus, Denmark; ^2^Department of Biomedicine, Faculty of Health, Aarhus University, Aarhus, Denmark; ^3^Comparative Medicine Lab, Department of Clinical Medicine, Faculty of Health, Aarhus University, Aarhus, Denmark

**Keywords:** cerebrospinal fluid, brain, spinal cord, lymphatic network, glymphatic system, aquaporin

## Abstract

A comprehensive understanding of the cerebrospinal fluid (CSF) system is essential for our understanding of health and disease within the central nervous system (CNS). The system of CSF refers to all components involved in CSF production, movement, and absorption. In recent years, extensive research has resulted in vastly improved understanding of the CSF system in health and disease. Yet, several aspects remain to be fully clarified, notably along the spinal cord as the preponderance of research has focused on the brain. This review briefly summarizes the CSF system and its implications for CNS diseases and highlights the knowledge gaps that require further research.

## Introduction

Renewed attention has come to the cerebrospinal fluid (CSF) system due to its importance for central nervous system (CNS) homeostasis. The CSF system constitutes a crucial role in the CNS as it provides mechanical protection, ensures homeostasis, and facilitates communication between the CNS and peripheral nervous system, lymphatic system, vascular system, and immune system (Damkier et al., 2013; Aspelund et al., 2015; Louveau et al., 2015; Adigun and Al-Dhahir, 2021). Yet, some aspects of the CSF system remain to be fully clarified, notably along the spinal cord. Of utmost importance is bridging the knowledge gap between the brain and the spinal cord regarding the controversies of a glymphatic system and a lymphatic network, and further to understanding how these complex relationships in the CSF system contribute to health and disease. This review aims to describe the theories underlying the CSF system in relation to neurological diseases in the brain and spinal cord. This will provide the basis for highlighting the knowledge gaps that should be addressed through further research.

## CSF Production and Absorption

**Figure 1:**
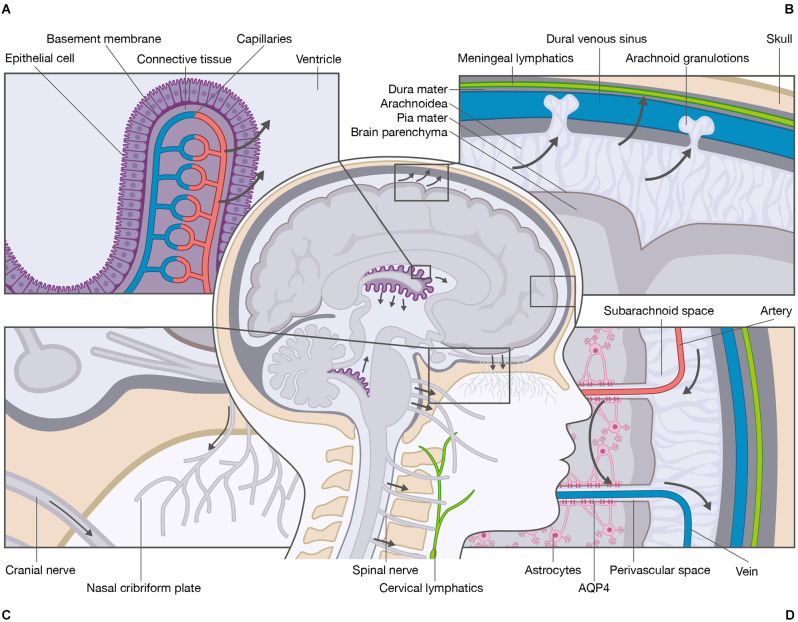
Schematic overview of the cerebrospinal fluid system. The primary site of cerebrospinal fluid (CSF) production is the choroid plexi located within the lateral, third, and fourth ventricles of the brain. **(A)** The choroid plexi consist of leaky epithelial cells, a basement membrane, connective tissue, and fenestrated capillaries. The CSF production is mediated by ionic transport that generates osmotic gradients and water transport from the blood system to the ventricular system. **(B)** Several anatomical sites are responsible for CSF absorption e.g., arachnoid granulations, meningeal lymphatics, and **(C)** cranial nerve sheaths and nasal cribriform plate to the cervical lymphatics. **(D)** One essential function of CSF is the delivery of nutrients and the removal of waste products. The most recently proposed mechanism for removal of waste is the glymphatic system. The CSF enters from the perivascular spaces surrounding arteries into the brain parenchyma *via* mechanisms that include AQP4 water channels located at the astrocytic end-feet. Within the brain parenchyma, CSF disperses and intermixes with the interstitial fluid (ISF) and waste products. The mixture of CSF, ISF, and waste products enters the perivascular spaces surrounding veins by unknown mechanisms, e.g., AQP4 water channels. From the perivascular spaces, the mixture leaves the brain parenchyma.

The CSF is a clear, colorless fluid that occupies the ventricular system, the cerebral and spinal subarachnoid spaces, and the perivascular spaces in the CNS. The fluid is a mixture of water, proteins at low concentrations, ions, neurotransmitters, and glucose that is renewed three to four times per day (Damkier et al., 2013; Hladky and Barrand, 2014; Spector et al., 2015). Several theories have been proposed to explain how CSF is produced. The classic theory states that the choroid plexi are the primary sources of CSF production. The choroid plexi develop from the ependyma protruding from the pia mater into the lateral, third, and fourth ventricles (Damkier et al., 2013; Hladky and Barrand, 2014). The plexi consist of a single layer of epithelial cells residing on a basement membrane, connective tissue, and fenestrated capillaries (Figure 1A). The epithelial cells are connected by tight junctions making the epithelial layer relatively tight, whereas the underlying fenestrated capillaries are relatively leaky. This enables the passage of compounds from the blood to the epithelial cells. The production of CSF depends on the transcellular movement of Na^+^ primarily driven by the Na^+^/K^+^-ATPase expressed at the luminal membrane facing the CSF. The movement of Na^+^ is accompanied by Cl^−^ and HCO_3_^−^ as well as water that follows the solute gradient. The water transport is distributed from the blood system to the ventricular system through aquaporin-1 (AQP1) water channels (Nielsen et al., 1993; Jensen et al., 2015). CSF is therefore not simply an ultrafiltrate of the blood, but a product of a tightly regulated ion transport that generates osmotic gradients and water transport. The production of CSF by the choroid plexi is believed to be relatively constant; however, the CSF secretion varies over the duration of a day with an average production of 650 ml and maximal production after midnight (Nilsson et al., 1992). The classic theory of CSF production has been challenged by findings in AQP1 knockout mice, demonstrating that water permeability across the choroid plexi is reduced by 85%, while the CSF secretion is only reduced by 35% (Oshio et al., 2005), suggesting other means of water transport across the epithelia. It is generally believed that the choroid plexi are the main sites of CSF production with contribution from extrachoroidal sites (Orešković and Klarica, 2010; Khasawneh et al., 2018); however, it has been proposed that the extrachoroidal sites are the main sites of CSF production with contribution from the choroid plexi (Orešković et al., 2017; Klarica et al., 2019).

As for CSF production, several theories of CSF absorption have emerged. The classic theory of CSF absorption states that absorption takes place from the subarachnoid spaces into the venous blood system through dural venous sinuses *via* cranial arachnoid granulations and into the lymph system *via* the nasal cribriform plate and the perineural sheaths (Klarica et al., 2019). Additional absorption is suggested to occur through cranial meningeal lymphatics embedded in the dura mater alongside arterial and venous vessels (Figures 1B,C; Aspelund et al., 2015; Jensen et al., 2015; Louveau et al., 2015; Tamura et al., 2020). Additional absorption has also been suggested to occur through spinal arachnoid granulations and spinal meningeal lymphatics (Chen et al., 2015b; Benveinste et al., 2017). Others have proposed that absorption through dural venous sinuses and/or lymphatics is of minor importance compared to absorption through blood microvessels (Klarica et al., 2019).

Considering the classic theory, the variation in CSF production must be matched by a similar variation in CSF absorption; otherwise, CSF accumulation would arise.

## CSF Movement

The CSF flow dynamics within the ventricular system and the subarachnoid spaces is thought to consist of two main types of movements: convective flow and pulsatile flow (Yildiz et al., 2017). Convective flow is a unidirectional motion from the choroid plexi in the lateral ventricles through the foramen of Monro into the third ventricle, passing through the cerebral aqueduct into the fourth ventricle. From the fourth ventricle, CSF exits the ventricular system through the three apertures where it enters the cerebral subarachnoid space, the spinal subarachnoid space, and the central canal of the spinal cord.

**Figure 2:**
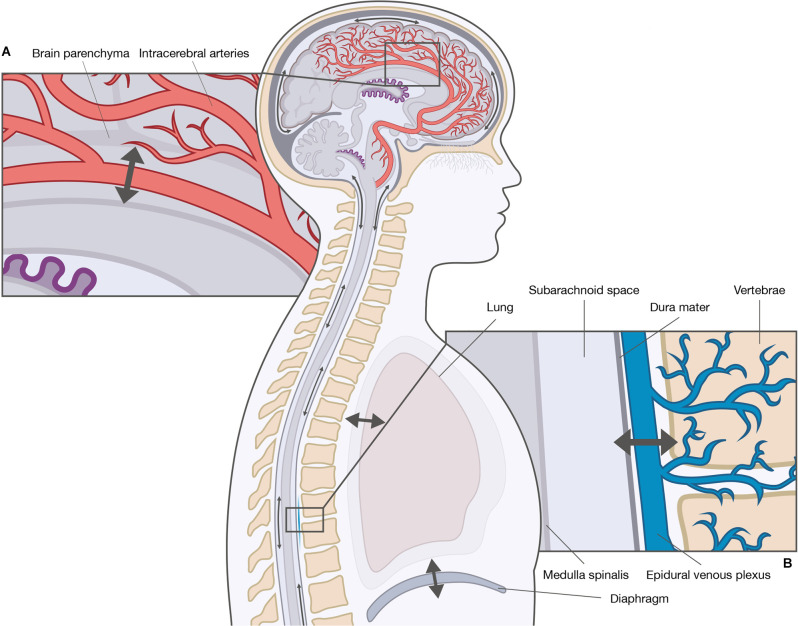
Driving forces of cerebrospinal fluid (CSF) movement within the ventricles and subarachnoid spaces. The movement of CSF is convective and pulsatile. The two main driving forces of the pulsatile CSF movement are the cardiac-driven force and the respiratory-driven force. These forces influence the arterial blood and the venous blood differently, and thereby CSF movement to a different extent. **(A)** The cardiac-driven force causes a change in blood volume leading to pulsations that are transmitted directly or indirectly to the CSF. **(B)** The respiratory force consists of thoracic respiration and diaphragmatic respiration. Both types of respirations influence the CSF movement through the venous system, e.g., epidural venous plexus by changes in the intrathoracic pressure.

The driving force of convective flow is thought to be the result of hydrostatic pressure gradients between the choroid plexi (high pressure) and arachnoid granulations (low pressure). The movement of CSF from the spinal subarachnoid space to the perivascular spaces and the lymph system is poorly described, although similar routes have been suggested (Chen et al., 2015b; Benveinste et al., 2017). The unidirectional movement has, however, been questioned by studies showing constant CSF movement, but without net unidirectional CSF displacement, suggesting a pulsatile flow (Orešković and Klarica, 2010; Klarica et al., 2019). Contrary to the unidirectional movement of the convective flow, the pulsatile flow is a bidirectional movement in upward (cranial) and downward direction (caudal) along the spinal cord, and in varying directions in the brain. Prior theories assumed the origin of the pulsatile CSF motion was the choroid plexi (Takizawa et al., 2018); however, to date, two main theories exist: the cardiac-driven theory and the respiratory-driven theory (Figure 2). The cardiac-driven theory states that changes in the blood volume are transmitted directly and indirectly to the CSF, i.e., a direct transmission of blood vessel pulsations to the CSF and an indirect transmission of blood vessel pulsations through other tissues to the CSF (Haughton and Mardal, 2014; Daouk et al., 2017). The respiratory-driven theory states that changes in the intrathoracic pressure are transmitted *via* the venous system to the CSF (Daouk et al., 2017; Aktas et al., 2019; Lloyd et al., 2020). Natural respiration may not contribute to the respiratory-driving force with the same magnitude as forced respiration. It is generally believed that inspiration elicits a cranial movement of CSF, while expiration elicits a caudal movement (Yamada et al., 2013; Chen et al., 2015a; Dreha-Kulaczewski et al., 2017; Aktas et al., 2019). However, both cranial and caudal CSF movements have been observed during inspiration as a result of epidural venous blood return to the thoracic spine from the cervical and lumbar spine (Lloyd et al., 2020). The relationship between the cardiac- and respiratory-driving forces is debated as the driving forces influence arterial and venous blood flow differently, thereby contributing to CSF movement to a different extent; however, the cardiac-driven force is thought to be responsible for the basic pulsatile CSF flow, while the respiratory-driven force is responsible for the large pulsatile CSF flow (Takizawa et al., 2017). The variability in the relative influence of the cardiac and respiratory forces has been attributed to variations in musculature and respiratory capacity (Yildiz et al., 2017), and the anatomical differences between the cranial and spinal cavity (Yildiz et al., 2017; Aktas et al., 2019; Lloyd et al., 2020). Yet, the exact relationship between the cardiac- and respiratory-driving forces remains to be fully clarified.

## Functions of The CSF System

An essential function of the CSF system is the maintenance of CNS homeostasis. As the CNS consists of highly active metabolic regions, waste products need to be cleared. The most recently proposed mechanism for waste clearance is the highly debated glymphatic system. The available literature primarily focuses on the mechanisms within the brain; thus, the mechanisms within the spinal cord remain largely elusive. The glymphatic system is a fluid conduit defined as an astrocyte-mediated fluid exchange of CSF and ISF in the brain (Iliff et al., 2012; Jensen et al., 2015; Rasmussen et al., 2018). Within the glymphatic system, CSF is thought to be driven from the subarachnoid space into the periarterial spaces surrounding penetrating arteries (Jensen et al., 2015), and along the periarterial spaces with a net convective flow following the direction of the blood flow (Mestre et al., 2018; Thomas, 2019; Kedarasetti et al., 2020). From the periarterial spaces, CSF enters the brain parenchyma. The water channels aquaporin-4 (AQP4) are expressed in the vascular endfeet of astrocytes (Trillo-Contreras et al., 2019) and have been implicated in the perivascular influx of water. AQP4 is a water channel with selective characteristics (Amasheh et al., 1995). The role of AQP4 is the most controversial part of the glymphatic system as convective transport through AQP4 is questionable from a physiological point of view. There is, however, consensus that CSF has a convective flow along the perivascular spaces of the larger blood vessels (i.e., arteries and arterioles) and diffusion across the smaller blood vessels (i.e., capillaries) situated at the neurovascular unit including AQP4. As discussed by others (Abbott et al., 2018), it is likely that a convective force increases the availability of CSF at the basal lamina in the capillaries, thereby adding to the diffusion. Within the brain parenchyma, CSF disperses and mixes with ISF. The compositions of CSF and ISF are comparable, but the major difference between the two compartments is that ISF is surrounded by an extracellular matrix, thereby enabling alteration in the fluid composition of for instance ions (Syková, 2008). Due to compositional differences in CSF and ISF, it is generally accepted that the two entities can be recognized as two compartments that communicate. The mixture of CSF, ISF, and waste products enters the perivenous space by mechanisms that are poorly understood (Iliff et al., 2012). From the perivenous space, the mixture leaves the brain by returning to the vein itself across the vessel wall or by returning to the CSF in the subarachnoid space (Figure 1D). From here, the CSF mixed with ISF components may leave the subarachnoid space *via* the meningeal lymphatic vessels, nerve sheaths, and nasal cribriform plate into the deep cervical lymph nodes (Iliff et al., 2012; Mestre et al., 2020).

The existence of a lymphatic network within the meninges of the brain was first described by the Italian anatomist Paolo Mascagni in 1787; a description that recently has been translated and published (Sandrone et al., 2019). With the rediscovery of the lymphatic network, findings demonstrate that the meningeal lymphatic vessels are embedded within the dura mater alongside arteries, veins, and cranial nerves (Aspelund et al., 2015; Jensen et al., 2015; Tamura et al., 2020). Here they create a network that facilitates waste clearance away from the brain and a direct link between the CNS and the peripheral immune system (Oliver et al., 2020). The entry of solutes and immune cells from the CSF to the meningeal lymphatics is proposed to occur through specific entry points along the vessels (Louveau et al., 2018).

Although considerable anatomical differences exist between the brain and the spinal cord, it seems reasonable to assume that the spinal cord has a waste clearance system and a lymphatic system resembling the systems proposed for the brain. In support of this assumption studies have demonstrated the existence of spinal perivascular spaces in rats (Lam et al., 2017; Liu et al., 2018), the existence of meningeal lymphatic vessels along the spinal cord in mice (Jacob et al., 2019), and expression of AQP1 and AQP4 water channels in the rodent spinal cord (Oklinski et al., 2014, 2016; Wei et al., 2017). As these findings are based upon animal studies, there is a need for human studies. These findings may have a vast impact on the emergence and progress of several CNS diseases, thus emphasizing the need for further research.

## The Relationship Between The CSF System and CNS Diseases

Knowledge of the CSF system has significant implications for understanding diseases in the brain and the spinal cord. It is, however, of great importance to acknowledge that any alteration in the CSF system may be influenced by other factors, e.g., aging, hypertension, atherosclerosis, and sleep deprivation (Benveinste et al., 2017), and that any alteration in the CSF system may influence other parts of the CSF system. A greater understanding of the relationship between the CSF system and CNS diseases may provide a better understanding of these diseases’ emergence and progress, and thereby reveal potential targets for treatment and intervention. A few examples of these relationships are given below.

### CNS Diseases Associated With Altered CSF Production

A physiological hyposecretion of CSF occurs with age due to increased amounts of connective tissue between the vasculature and the epithelial cells (Preston, 2001). This physiological age-dependent hyposecretion is thought to be additive to the changes in the brain’s waste clearance during the progression of dementia and Alzheimer’s disease (AD) as mentioned later in the text. A pathophysiological hypersecretion of CSF is relatively rare and has mostly been described in connection to choroid plexus papillomas or neoplasms (Crawford and Isaacs, 2019; Crea et al., 2020). Yet, a common complication of subarachnoid hemorrhage is hydrocephalus (Chen et al., 2017). This type of hydrocephalus was previously believed to be caused by an obstruction of the CSF flow in the cerebral aqueduct or the arachnoid granulations; however, recent studies suggest that hemorrhage causes an inflammation-dependent hypersecretion of CSF by the choroid plexi (Karimy et al., 2017; Li et al., 2018). Knowledge of the mechanisms leading to hemorrhage-dependent hypersecretion could provide targets for inhibition of CSF secretion following subarachnoid hemorrhage.

### CNS Diseases Associated With Altered CSF Clearance and Absorption

The progression of neurodegenerative diseases e.g., AD has been linked to attenuation of the waste clearance system. AD is an age-dependent disease marked by the accumulation of specific proteins, neurofibrillary tangles, and amyloidß peptide, in the brain. These proteins are proposed to be cleared by the waste clearance system, thus reduced movement of CSF from the periarterial spaces to the brain parenchyma *via* AQP4 could facilitate protein accumulation in the brain (Rasmussen et al., 2018; Oliver et al., 2020). Supportive of this assumption, a study of human AD brains found that loss of AQP4 localized to the perivascular astrocytic endfeet was associated with AD (Zeppenfeld et al., 2017; Reeves et al., 2020). Altered AQP4 expression has been linked to the formation of edema following CNS injury (Sun et al., 2003; Nesic et al., 2006; Zhang et al., 2015). An early down-regulation and a late up-regulation of APQ4 expression have been found to correlate with an increased water content within spinal cord injured rats (Nesic et al., 2006).

As described previously, lymphatic vessels are essential for fluid balance and immune surveillance in tissues (Oliver et al., 2020), thus alterations in the lymphatic vessels may facilitate fluid imbalance and CNS-directed immune responses. These considerations have been addressed in mice with chemical-induced spinal cord injury, where the authors found spinal cord injury to cause lymphangiogenesis, which exacerbated immune-cell infiltration and demyelination of the spinal cord concomitant with reduced regeneration (Jacob et al., 2019). The CNS-directed immune responses have also been addressed in mice with experimental autoimmune encephalomyelitis, demonstrating that lymphatic ablation attenuated the immune response of reactive immune cells around demyelinated lesions (Louveau et al., 2018). The relationship between CNS diseases and CNS-directed immune responses as well as fluid imbalance facilitated by the lymphatic vessel may also have implications for other CNS diseases e.g., traumatic spinal cord injury.

### CNS Diseases Associated With Altered CSF Movement

Disturbances in the CSF movement may influence the functioning of the waste clearance system. Thus, CNS diseases causing an obstruction in the brain or along the spinal cord, and thereby CSF movement disturbances, may promote the emergence and progress of secondary CNS diseases. This is demonstrated by the association between traumatic spinal cord injury and posttraumatic syringomyelia: a disease characterized by the formation of fluid-filled cysts within the spinal cord parenchyma (Vandertop, 2014). In mice suffering from posttraumatic syringomyelia, an increased AQP4 expression has been found, suggesting increased AQP4 expression as a driver of cyst formation (Hemley et al., 2013). Supportive of this finding, studies of traumatic spinal cord injured rats found increased APQ4 expression to be correlated with water content within the spinal cord (Nesic et al., 2006; Pan et al., 2019). Thus, increased AQP4 expression might be implicated in CNS diseases with excessive parenchymal fluid accumulation; however, the exact mechanisms remain elusive.

As the preponderance of studies investigate the mechanisms of the CSF system and its implications for neurological diseases in the brain, our understanding is sparse when it comes to the mechanisms in the spinal cord. It does, however, seem reasonable to believe that the assumptions made in the brain, to some extent, are applicable to the spinal cord. Yet, the examples above highlight the complexity of the CSF system. Therefore, research is needed to evaluate the CSF system in a more integrative view to elucidate how changes in one part of the system lead to changes in other parts of the system.

## Conclusion

Despite significant advances in our understanding of the CSF system, many processes remain to be elucidated. Notably, as the majority of studies focuson the brain, there is a significant knowledge gap regarding the spinal cord. Both the anatomical and physiological differences between the brain and spinal cord hamper the translation of findings found in the brain to the spinal cord, thus more research into the mechanisms in the spinal cord is warranted.

## Author Contributions

All authors contributed to conception and design of the work. TW and HD wrote sections of the manuscript. All authors contributed to the article and approved the submitted version.

## Conflict of Interest

The authors declare that the research was conducted in the absence of any commercial or financial relationships that could be construed as a potential conflict of interest.

## Publisher’s Note

All claims expressed in this article are solely those of the authors and do not necessarily represent those of their affiliated organizations, or those of the publisher, the editors and the reviewers. Any product that may be evaluated in this article, or claim that may be made by its manufacturer, is not guaranteed or endorsed by the publisher.
